# Description of a New Species of Caliscelidae from the High Altitude Region of Xizang Based on Morphological and Molecular Evidence †

**DOI:** 10.3390/insects17070667

**Published:** 2026-06-25

**Authors:** Helin Chan, Muye Niu, Zhi Huang, Xiujuan Liu, Jiancheng Zang

**Affiliations:** 1 College of Plant Science, Xizang Agriculture and Animal Husbandry University, Linzhi 860000, Chinaniumy20260406@163.com (M.N.);; 2 Laboratory of Resource and Applied Insect in the Xizang Plateau, Linzhi 860000, China

**Keywords:** new species, Hemiptera, Caliscelidae, COI gene, phylogenetic tree, Xizang

## Abstract

This study describes a new species of the family Caliscelidae (Hemiptera) from the Xizang region of China—*Peltonotellus lasaensis* sp. nov. Through integrated morphological examination (including external morphology and genitalia dissection), and mitochondrial gene (COI) sequence analysis, the taxonomic status of this species was determined. Morphological results reveal that the male aedeagus terminates in valvular lobes, with ventral processes comprising a pair of spinous projections. In females, the type IX arcuate process bears approximately 15 ridge teeth on the dorsal margin, a feature that distinctly differentiates it from congeners. Molecular phylogenetic analysis based on COI sequences places the new species within a well-supported clade of Caliscelidae, confirming its genus-level placement in *Peltonotellus*. This study represents the first record of Caliscelidae in the high-altitude regions of Xizang (3650–3800 m), thereby enriching the diversity data of Caliscelids in Asia and providing valuable insights into the biogeography of the Qinghai-Xizang Plateau.

## Simple Summary

The family Caliscelidae, a member of the superfamily Fulgoroidea within Hemiptera, currently includes 81 genera and 271 valid species. However, Caliscelidae in high-altitude regions remains poorly documented, especially in the distribution and taxonomic information of the Qinghai-Xizang Plateau, which is extremely scarce. In this paper, we provide a description of a new species of Caliscelidae from the Xizang. Characters important for morphological identification and mitochondrial gene sequence analysis are discussed and illustrated.

## 1. Introduction

Hemiptera, one of the most diverse insect orders, is globally distributed across various ecosystems and plays a significant role in agricultural and forestry systems [1]. The family Caliscelidae (superfamily Fulgoroidea) has attracted taxonomic interest due to its distinctive morphology and complex speciation patterns [2]. Members of this family predominantly inhabit tropical and subtropical regions, and the family currently includes 81 genera and 271 valid species worldwide. However, taxonomic studies in Asia remain limited. The Xizang region of southwestern China, a recognized biodiversity hotspot owing to its unique topography and ecosystems, harbors poorly documented Caliscelidae diversity, with numerous undescribed species likely awaiting discovery. Recent advances in molecular systematics have complemented traditional taxonomy, particularly through mitochondrial cytochrome oxidase subunit I (COI) gene analysis, which enables precise delineation of genetic divergence among closely related species and validation of morphological classifications [3]. Phylogenetic reconstructions of Fulgoroidea using COI have suggested revisions for several genus-level taxa. Molecular data for Asian Caliscelidae are still relatively scarce, with only a limited number of COI sequences available for species from this region. This limits comprehensive insights into their evolutionary history and biogeographic distribution.

Xizang, situated in southwestern China, harbors a remarkable diversity of Hemiptera insects owing to its unique topography and diverse climatic zones [4]. However, studies on the family Caliscelidae in this region have long been neglected due to challenges in specimen collection and limited taxonomic resources. The Caliscelidae fauna of China was comprehensively treated by Zhang et al. [5] in *Fauna Sinica*, providing a critical taxonomic baseline for the region. Previous research has demonstrated that male genital structures (e.g., aedeagus morphology and clasper characteristics) in this family exhibit pronounced interspecific variation, providing key diagnostic traits for species delimitation [6]. Furthermore, geographical isolation and microhabitat specialization likely exacerbate species differentiation in this region. Thus, an integrative approach combining morphological and molecular data will be essential for elucidating the diversity and evolutionary drivers of this understudied group.

This study examined specimens of the family Caliscelidae collected from the Xizang region in southwestern China through comprehensive morphological analysis (including external morphology and genitalia dissection) and molecular characterization using COI gene sequencing. As a result, we describe a new species, *Peltonotellus lasaensis* sp. nov. The study objectives were threefold: (1) to provide detailed morphological descriptions and high-resolution diagnostic images of the new species; (2) to reconstruct a phylogenetic tree based on COI sequences to determine its taxonomic position; and (3) to evaluate the biodiversity conservation significance of Caliscelidae in the Xizang region.

## 2. Materials and Methods

### 2.1. Specimen Collection and Preservation

The specimens were collected from plantation habitats in Xizang, southwestern China, at coordinates 29°38′20″ N, 91°9′23″ E and 29°41′38″ N, 91°5′38″ E, with an elevation range of 3650–3800 m above sea level. Field collections were conducted from May to September 2023 by Huang Zhi using two complementary methods: (1) sweep netting of vegetation and (2) Malaise traps. All adult specimens were immediately preserved in 95% ethanol upon collection to maintain DNA integrity for molecular analysis. Voucher specimens are currently deposited in the entomological collection of the Plateau Resource Insects and Applied Entomology Laboratory at Xizang Agricultural and Animal Husbandry University for long-term preservation and future reference.

### 2.2. Morphological Observation and Imaging

Morphological examinations were conducted using a Leica M205 C stereomicroscope (Leica Microsystems, Wetzlar, Germany), with body length (measured from the head apex to wing tip) and maximum head width recorded as mean ± standard deviation [7]. For male genitalia preparation, abdomens were immersed in 10% KOH solution and heated in an 80°C water bath for 30 min [8], followed by thorough rinsing with distilled water. The cleared specimens were then transferred to glycerol, where the aedeagus and claspers were carefully separated using microdissection needles for observation. All microscopic images were captured using the Leica M205 C system, with photographic documentation including multiple focal planes and views (dorsal, lateral, and ventral) of key morphological structures. The images were subsequently processed using Adobe Photoshop 2023 software, with scale bars calibrated against the microscope’s integrated measurement ruler.

### 2.3. Molecular Experiment

**DNA Extraction and PCR Amplification:** Genomic DNA was extracted from whole worm tissues using a magnetic-bead-based DNA extraction kit (Wuhan Tianyi Huayu Gene Technology Co., Ltd., Wuhan, China), with nucleic acid purity, concentration, and integrity assessed through NanoDrop spectrophotometry (Thermo Scientific, Waltham, MA, USA) and agarose gel electrophoresis (Beijing Liuyi Biotechnology Co., Ltd., Beijing, China). The mitochondrial COI gene was amplified using universal insect primers (Table 1) following standard barcoding protocols [9,10], with all PCR reactions performed in duplicate for bidirectional sequencing. The resulting sequences were assembled and queried against the NCBI nr/nt database, where the top 10 matching sequences (e.g., MW928530.1) were retained as reference materials alongside the highest-scoring match for species identification. The newly obtained sequence has been deposited in GenBank under accession number PRJNA1253866.

**Phylogenetic Analysis:** COI sequences of 12 Caliscelidae species were obtained from published mitochondrial genomes [11] and used as the ingroup. Four Issidae species were selected as outgroup taxa. All sequences were aligned using the ClustalW algorithm in MEGA 11. A maximum likelihood (ML) tree was constructed with 1000 bootstrap replicates. The resulting phylogenetic tree was visualized and annotated using FigTree v1.4.4.

## 3. Results

***Peltonotellus lasaensis* sp. nov.**

Chinese common name: 拉萨敏杯瓢蜡蝉.

### 3.1. Morphological Description (Figure 1, Figure 2 and Figure 3)

**Type Materials**

**Holotype: ♂:** Lhasa City, Xizang Autonomous Region, China; the middle and lower reaches of the Lhasa River valley; 29°38′6″ N, 91°8′49″ E; elevation 3600–3700 m; 10 July 2024; Lab of Resource and Applied Insect in the Xizang Plateaus, Chan Helin.

**Paratypes: ♀:** Lhasa City, Xizang Autonomous Region, China; forest restoration area on a mountainside near the Lhalu Wetland National Nature Reserve, 29°41′38″ N, 91°5′38″ E; elevation 3600–3700 m; 15 July 2023; Lab of Resource and Applied Insect in the Xizang Plateaus, Huang Zhi.

**Figure 1:**
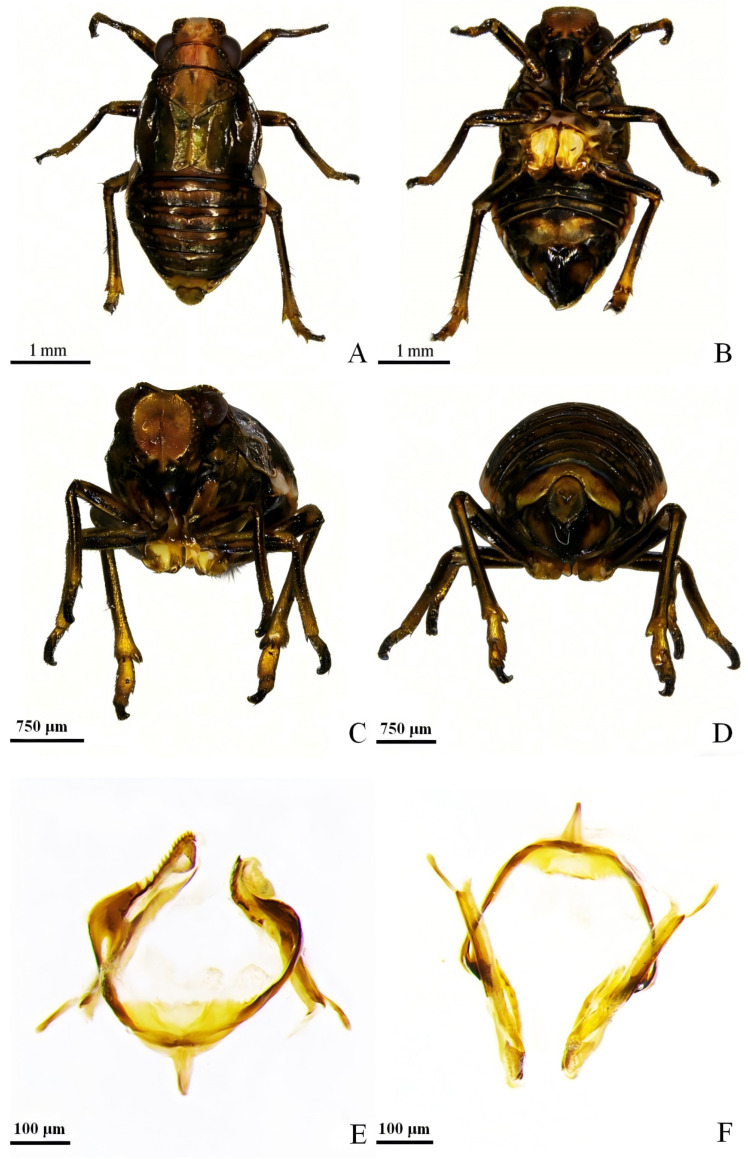
**♀ of *Peltonotellus lasaensis* sp. nov.** (**A**) dorsal view; (**B**) ventral view; (**C**) frontal view; (**D**) caudal view; (**E**) type IX arcuate process and gonapophyseal bridge, dorsal view; (**F**) same, frontal view.

**Figure 2:**
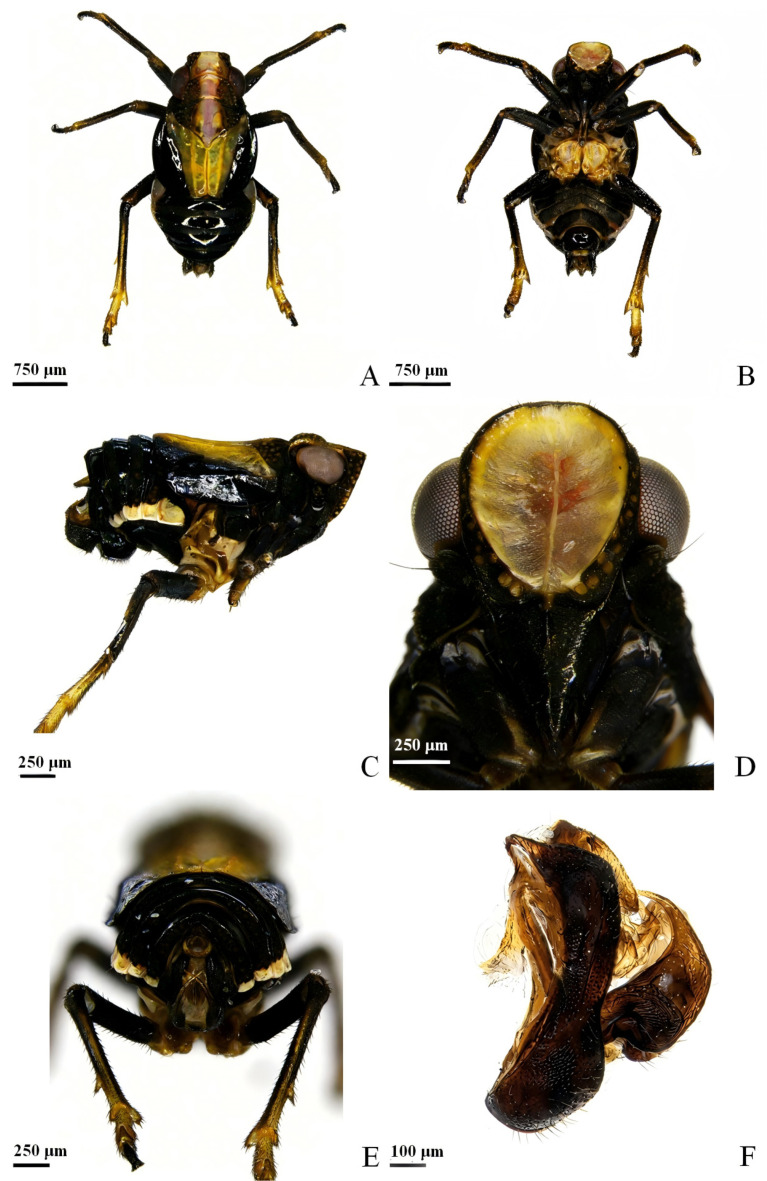
**♂ of *Peltonotellus lasaensis* sp. nov.** (**A**) dorsal view; (**B**) ventral view; (**C**) lateral view; (**D**) head; (**E**) tail; (**F**) genital segment, lateral view.

**Figure 3:**
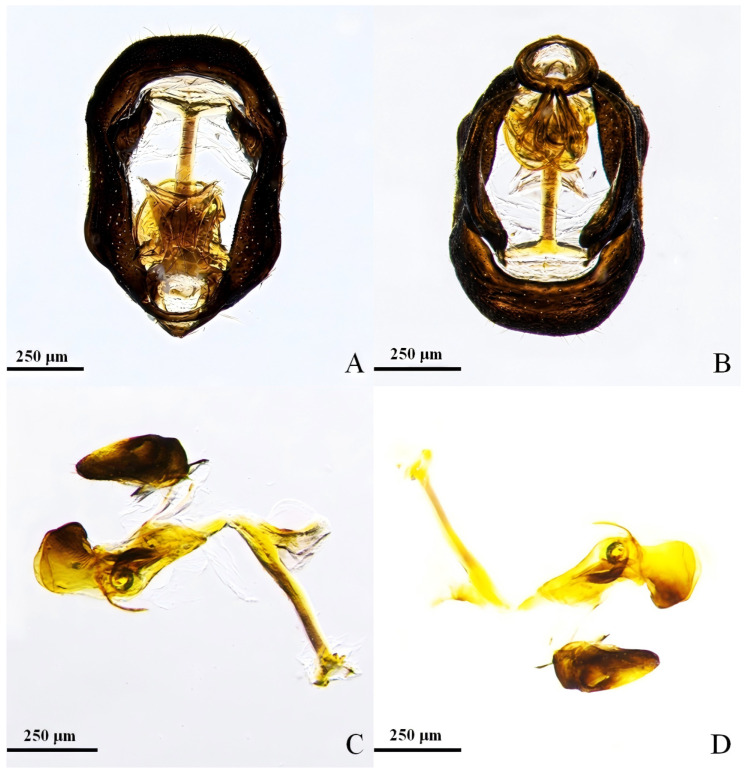
**♂ of *Peltonotellus lasaensis* sp. nov.** (**A**) genital segment, inner view; (**B**) genital segment, dorsal view; (**C**) aedeagus, view 1; (**D**) aedeagus, view 2.

**Etymology**

The specific epithet *lasaensis* is derived from Lhasa, the type locality in the Xizang Autonomous Region, China.

**Description**

Body length: male 3.0–3.1 mm (*n* = 5) (Figure 2A–E), female 3.3–3.7 mm (*n* = 4) (Figure 1A–D).

Body color sexually dimorphic. Male: head white, median pronotal region white bordered with reddish margins, lateral portions black with irregular yellowish-brown spotting. Compound eyes dark brown. Forewings translucent, costal margin yellowish, remaining part blackish. Abdomen ventrally uniformly black. Female: head and pronotum predominantly brown, with irregular yellowish-brown lateral spotting. Compound eyes dark brown. Forewings semi-transparent, uniformly brown. Abdomen ventrally brown, with irregular black patches.

Head (Figure 1C and Figure 2D): Wider than long (male width 0.64 ± 0.02 mm, female 0.77 ± 0.02 mm). Compound eyes elliptical. Antennae short; scape distinctly swollen, with yellowish-brown punctate markings; flagellum slender throughout.

Thorax (Figure 1A and Figure 2A): Pronotum trapezoidal, in male with pronounced apical convergence forming distinct angle. Mesoscutum triangular, with well-defined central longitudinal depression. Forewings with prominent venation; in male with striking color demarcation between costal and posterior regions.

Male genitalia (Figure 2E,F and Figure 3A–D): Paramere nearly rectangular in lateral view; ventral posterior margin bluntly rounded, prominently protruding; dorsal process strongly curved, terminating in sharp point. Anal segment circular, basal margin slightly thickened, distal protrusion present; anal orifice positioned centrally. Caudal segment opening pod-shaped posteriorly. Aedeagus a shallow U-shaped tubular structure, distally divided into valvular lobes; ventral processes a pair of elongated spine-like projections.

Female genitalia (Figure 1D–F). Type IX arcuate process bearing approximately 15 distinct ridge teeth along dorsal margin. Posterior connective plate with minimally protruding denticles along outer margin.

### 3.2. Phylogenetic Tree

**Figure 4:**
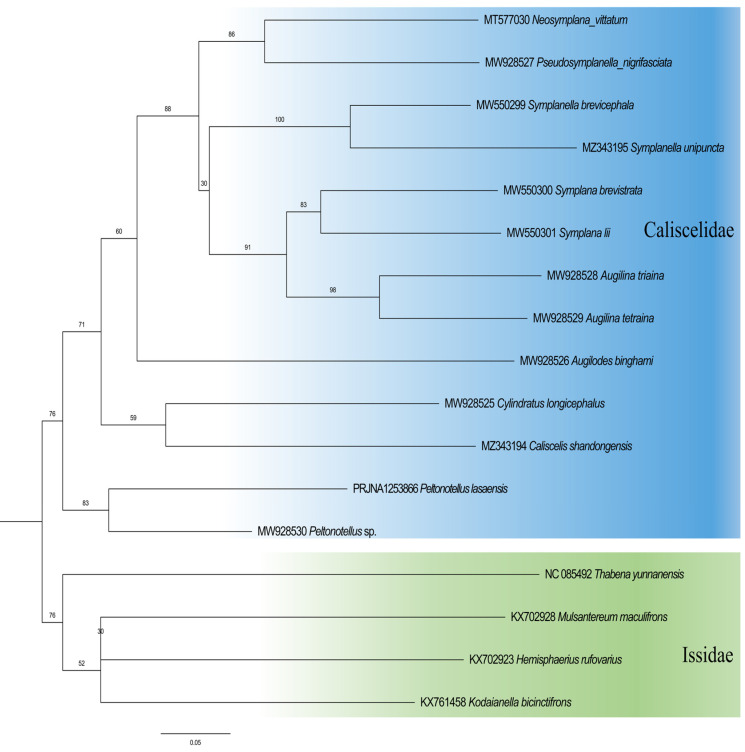
Phylogenetic tree constructed based on COI sequences.

**Figure 5:**
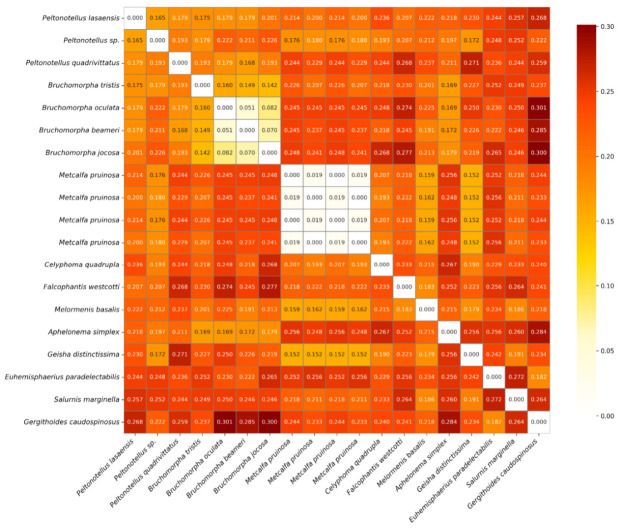
Genetic distance heatmap based on COI sequences. Note: the greater the genetic distance, the darker the color and the larger the value.

The ML tree based on COI sequences (Figure 4) places *Peltonotellus lasaensis* sp. nov. within the family Caliscelidae, forming a well-supported clade with *Peltonotellus* sp. from GenBank (bootstrap value > 97%). This clade is nested within a larger assemblage of Caliscelidae genera, consistent with current classification. The genetic distance (K2P) between *P. lasaensis* sp. nov. and its closest congener is 16.5%, substantially exceeding the typical species-level threshold of 2% [12], further supporting its recognition as a distinct species (Figure 5) [13].

### 3.3. Differential Diagnosis

The discovery of *P. lasaensis* at 3800 m elevates the altitudinal distribution limit of Caliscelidae; contrasting with *P. niger* (2347–3147 m) and *P. brevis* (unspecified). The female genital ridge tooth count exhibits: *P. lasaensis* (15 teeth) > *P. niger* (11 teeth) > *P. brevis* (10 teeth). The male aedeagus of *P. lasaensis* displays a unique valvular lobes + paired spinous processes combination, distinct from *P. niger*’s dual processes (thin process + transverse process) and *P. brevis*’s single short process. These three species demonstrate NW–SW geographic isolation: Ningxia (*P. brevis*) → Gansu (*P. niger*) → Xizang (*P. lasaensis*). Based on data from Meng et al. (2015) and this study, Table 2 compares morphological characteristics of three Chinese new species in *Peltonotellus* [14].

Diagnosis: *Peltonotellus lasaensis* sp. nov. differs from its Chinese congeners by the following combination of characters:

(1) Male aedeagus distally divided into valvular lobes, with a pair of elongated spine-like ventral processes (vs. single short thin process directing to base at right side in *P. brevis*; short thin process plus short transversal process in *P. niger*; apex with a pair of large laterally directed lobes in *P. lobulus*; simple tubular, without any process in *P. albulus*; aedeagal internal structure not described in detail for *P. fasciatus* and *P. quadrivittatus*, but both species clearly differentiated by body coloration).
(2) Female type IX arcuate process with approximately 15 distinct ridge teeth along dorsal margin (vs. approximately 10 teeth in *P. brevis*; approximately 11 dorsal teeth plus six small lateral teeth in *P. niger*; tooth count not described in original descriptions for *P. lobulus* and *P. albulus*; structure not described for *P. fasciatus* and *P. quadrivittatus*).
(3) Body larger: male 3.0–3.1 mm, female 3.3–3.7 mm (vs. male 1.7–1.9 mm, female 2.8–3.0 mm in *P. brevis*; male 2.2–2.4 mm, female 2.5–2.7 mm in *P. niger*; male 2.6 mm, female 3.1 mm in *P. lobulus*; male 2.3 mm, female 2.8 mm in *P. albulus*).
(4) Coloration sexually dimorphic: male head white with reddish margins and lateral black spots, forewings translucent with distinct costal yellowish and posterior blackish gradient; female predominantly brown with irregular black abdominal patches (vs. body black with white median stripe in *P. niger*; body milky white in *P. albulus*; pale longitudinal median stripe from vertex to abdominal apex plus black round spot on each side of frontal median carina in *P. fasciatus*; vertex with four longitudinal dark stripes in *P. quadrivittatus*) [15].

## Figures and Tables

**Table 1:** Primer information.

Site name	Primer name	Primer sequence	Product size	TM value
COI-KC	LCO1490	GGTCAACAAATCATAAAGATATTGG	650	42
	HC02198	TAAACTTCAGGGTGACCAAAAAATCA		

**Table 2:** Comparing morphological characteristics of three Chinese new species in *Peltonotellus*.

Character	*P. brevis* Meng et al., 2015 (Ningxia)	*P. niger* Meng et al., 2015 (Gansu)	*P. lasaensis* Chan et al., 2025 (Xizang)
Type locality	Tongxin County, Ningxia (altitude unspecified)	Luqu County, Gansu (2347–3147 m)	Lhasa, Xizang (3650–3800 m)
Body length (mm)	♂ 1.7–1.9 ♀ 2.8–3.0	♂ 2.2–2.4 ♀ 2.5–2.7	♂ 3.0–3.1 ♀ 3.3–3.7
Key coloration			
Male	Head/thorax with broad white median stripe; forewings dark brown/orange	Entirely black; white median stripe on head/thorax	Head white + red margins; lateral black spots; forewings translucent (yellow-black gradient)
Female	Light yellowish-brown; abdominal B&W stripes	Dark fulvous; black spots flanking white stripe	Predominantly brown; irregular black abdominal patches
Male genitalia			
Aedeagus structure	Short thin process on right side (not reaching phallobase base)	Short thin process + transverse process on right	Distally divided into valvular lobes; paired spinous ventral processes
Phallobase dorsal margin	Concave near distal 1/3	Slightly concave	U-shaped tubular
Female genitalia			
Teeth on type IX arcuate process	~10 teeth on dorsal margin of posterior connective lamina	~11 dorsal teeth + 6 small lateral teeth	~15 ridge teeth (key diagnostic)
Denticle protrusion	Minimal	Prominent	Distinct
Head ratio (width/length)	♂ 1.1×, ♀ 0.9×	0.8×	Width > length (♂ 0.64 mm, ♀ 0.77 mm)
Forewing features	Grayish-brown; no transparency	Subtransparent; prominent veins	Semi-transparent; ♂ color-demarcated, ♀ uniformly brown
Habitat	Mountain vegetation	High-altitude grasslands	One of the highest recorded altitudes for the family (3650–3800 m)

## Data Availability

The data presented in this study are available on request from the corresponding author.
